# Simultaneous Screening of 24 Target Genes of Foodborne Pathogens in 35 Foodborne Outbreaks Using Multiplex Real-Time SYBR Green PCR Analysis

**DOI:** 10.1155/2010/864817

**Published:** 2010-09-28

**Authors:** Hiroshi Fukushima, Jun Kawase, Yoshiki Etoh, Kumiko Sugama, Shunshuke Yashiro, Natsuko Iida, Keiji Yamaguchi

**Affiliations:** ^1^Shimane Prefectural Institute of Public Health and Environmental Science, 582 Nishihamasada, Matsue, Shimane 690-0122, Japan; ^2^Fukuoka Institute of Health and Environmental Sciences, 39 Mukaizano, Dazaifu, Fukuoka 818-0135, Japan; ^3^Fukushima Institute of Public Health, 16-6 Houkida-aza-mitouchi, Fukushima 960-8560, Japan; ^4^Kumamoto Prefectural Institute of Health and Environmental Science, 1240-1 Kurisaki, Udo, Kumamoto 869-0425, Japan; ^5^Shizuoka Institute of Environment and Hygiene, 4-27-2 Kitayasuhigashi, Aoi, Shizuoka 420-8637, Japan; ^6^Hokkaido Institute of Public Health, West 12, Nortb 19, North Ward, Sapporo, Hokkaido 060-0819, Japan

## Abstract

A set of 8 multiplex real-time SYBR Green PCR (SG-PCR) assays including 3 target primers and an internal amplification control (IAC) primer was simultaneously evaluated in 3 h or less with regard to detection of 24 target genes of 23 foodborne pathogens in 7 stool specimens of foodborne outbreak using a 96-well reaction plate. This assay, combined with DNA extraction (QIAamp DNA Stool Mini kit), offered detection of greater than 10^3^-10^4^ foodborne pathogens per g in stool specimens. The products formed were identified using melting point temperature (*Tm*) curve analysis. This assay was evaluated for the detection of foodborne pathogens in 33 out of 35 cases of foodborne outbreak, using 4 different PCR instruments in 5 different laboratories. No interference from the multiplex real-time SG-PCR assay, including IAC, was observed in stool specimens in any analysis. We found multiplex real-time SG-PCR assay for simultaneous detection of 24 target genes of foodborne pathogens to be comprehensive, rapid, inexpensive, accurate, of high selectivity, and good for screening probability.

## 1. Introduction

Technological advances in the past 2 decades have substantially increased the possibility of rapid diagnostic testing for many diseases.However, for bacterial pathogens which cause foodborne infections or foodborne outbreaks, traditional culture methods, which can take up to 1 week, are still the only method many microbiology laboratories routinely use for diagnosis [1]. Real-time PCR is one of the principle methodologies emerging for rapid diagnosis of foodborne outbreak. We previously reported a duplex real-time SYBR Green PCR (SG-PCR) screening system of 8 specific genes of foodborne pathogens in 5 fecal samples [2–4]. The real-time SG-PCR assay combined with DNA extraction using a QIAamp DNA Stool Mini kit offered detection of greater than 10^3^-10^4^ foodborne pathogens per g in fecal samples. For diagnosis and management of foodborne outbreaks, this could distinguish patients infected with foodborne pathogens from healthy carriers. The introduction of this screening system in foodborne outbreak investigations provides an opportunity for comprehensive and rapid detection of pathogens in fecal samples. The results can quickly inform a public health administrator about the causative pathogens of foodborne outbreak, allowing a more accurate, effective and timely response. If it is possible to test for almost all foodborne pathogens including enteric and toxin-producing bacteria at a time, real-time PCR tests will certainly be useful for multiplex screening of foodborne pathogens. With multiplex PCR tests, if multiple bacteria could be simultaneously detected in the same reaction tube or during the same run, molecular diagnosis may prove to be very cost-effective. However, at present, published evaluations of these assays are insufficient.

One of the risks associated with testing samples by PCR is the occurrence of a false negative resulting from PCR inhibition [5, 6]. While positive and negative controls are normally run with every PCR master mix to ensure integrity of the reagents, PCR inhibition by the sample matrix can prevent amplification of the target template, resulting in false-negative reporting [5, 6]. Therefore, it is necessary to include an internal amplification control (IAC) in each individual reaction mixture to prevent reporting of false negatives [5]. Previous works have utilized various methods for developing and using an IAC for detection of a single target gene, except in the case of 4-target TaqMan multiplex PCR to detect *V. parahaemolyticus* [7]. 

The objective of the present study was to establish simple and specific methods to simultaneously detect 24 specific genes of foodborne pathogens in 7 stool specimens in a real-time SG-PCR assay using a 96-well reaction plate containing a universal, noncompetitive IAC.

## 2. Materlal and Methods

### 2.1. Bacterial Strains

The 659 foodborne pathogens used in this study are shown in Table 2. The 23 foodborne pathogens (enteroinvasive *Escherichia coli*, enteropathogenic *E. coli*, enterohemorrhagic *E. coli*, enterotoxigenic *E. coli*, enteroaggregative *E. coli,* diffusively adherent *E. coli*, *Shigella *spp., *Salmonella *spp., *Yersinia enterocolitica*, *Y. pseudotuberculosis*, *Providencia alcalifaciens*, *Plesiomonas shigelloides*, *Campylobacter jejuni*,* C. coli*, *Vibrio cholerae*, TDH-positive *V. parahaemolyticus*, TRH-positive *V. parahaemolyticus*, *Aeromonas hydrophila*, *Staphylococcus aureus*, emetic *Bacillus cereus,* enterotoxigenic *B. cereus, Clostridium perfringens, *and *Listeria monocytogenes*) described as control strain in Table 2 are used as control for PCR analysis. DNA was isolated from cultured bacteria to test the specificity of the primers used in this study. Viable counts were obtained by culturing each dilution (10 *μ*L) overnight at 37°C on tryptic soy agar (TSA) plates for aerobic bacteria and TSA plates containing 3% NaCl for *Vibrio *spp. *Yersinia *spp. strains were cultured at 28°C for 48 h. The *Clostridium perfringens *strains were cultured on TSA overnight at 37°C using anaerobic conditions. The *Campylobacter jejuni *strains were cultured at 37°C for 48 h on Skirrow agar plates under microaerobic conditions.

### 2.2. Internal Amplification Control (IAC) and IAC Primers for PCR

An IAC was included in the assay by adding a small amount of PCR products using IAC primers from the bacterium *Yersinia ruckeri* (JCM15110), which is the causative agent of enteric red-mouth disease in salmonid fish species [8] and the presence of this bacterium in human fecal samples and food samples is never reported. Bacterium used for DNA extraction was grown on brain heart infusion broth (Difco) at 30°C for 2 days. Two IAC primer pairs with different *Tm* of PCR products were used for amplifying 16S rRNA gene (GenBank accession no. X75275) of *Y. ruckeri*. One IAC primer was* yers* described by Lund et al. [9] and the *Tm* value of PCR product used for this primer was 77.3 ± 0.15°C. Another IAC primer sequence of *yersH2*-F and* yersH2*-R were chosen by alignment of 16S rRNA gene sequence from foodborne pathogens shown in Table 1 using the BLAST program within GenBank and was designed by Biosearch Technologies Inc. The *Tm* value of PCR product used for this primer was 86.0 ± 1.5°C.

### 2.3. DNA Extraction

For the DNA isolation from bacterial cultures, one milliliter of broth culture was centrifuged at 12,000 × *g *for 3 minutes. The pellet was then washed in 1 mL of distilled water, centrifuged, and suspended into 1 mL of distilled water. Each 200 microliters of suspension, containing 10^8^ foodborne bacterial cells, was treated with the QIAamp DNA Stool Mini kit (Qiagen) according to manufacturer instructions. DNA preparations were used immediately for PCR amplification and stored at −20°C. Four *μ*L of DNA sample were used for PCR assay. For the DNA isolation from stool samples, stool samples (1 g) were weighed aseptically, placed into sterile tubes, and homogenized with 9 mL of distilled water. Two-hundred *μ*L of this stool suspension was treated with the QIAamp DNA Stool Mini kit according to manufacturer instructions in 1 h or less.

### 2.4. Primers

Primers were used for 24 specific genes of 23 foodborne pathogens which belonged to 16 species:* Escherichia coli* (enteroinvasive *E. coli*, enteropathogenic *E. coli*, enterohemorrhagic *E. coli*, enterotoxigenic *E. coli*, enteroaggregative *E. coli, *and diffusively adherent *E. coli*), *Shigella *spp., *Salmonella *spp., *Yersinia enterocolitica*, *Y. pseudotuberculosis*, *Providencia alcalifaciens*, *Plesiomonas shigelloides*, *Campylobacter jejuni*,* C. coli*, *Vibrio cholerae*, *V. parahaemolyticus* (TDH-positive and TRH-positive types), *Aeromonas hydrophila*, *Staphylococcus aureus*, *Bacillus cereus* (emetic and enterotoxigenic types)*, Clostridium perfringens, *and *Listeria monocytogenes,* and the 2 IAC primers are listed in Table 1. The size and melting point temperature (*Tm*) values of PCR products are also listed in Table 1. The specificity and sensitivity of PCR assay using each primer were confirmed in each referred report. The primer pairs of tdh-F176 and tdh-R422 for the detection of *tdh*-positive *V. parahaemolyticus*, yadA-F1757 and yadA-R1885 for the detection of *Y. enterocolitica* and *Y. pseudotuberculosis*, PSG-F64 and PSG-R313 for the detection of *P. shigelloides*, ipaH1672-F and ipaH1761-R for the detection of *Shigella* spp., and EIEC, daaD-F31 and daaD-R263 for the detection of DAEC were chosen by alignment of virulent or specific gene sequences from foodborne pathogens shown in Table 2 using the BLAST (Basic Local Alignment Search Tool) program within GenBank and was designed by Biosearch Technologies Inc. (Tokyo). The *Tm *values of these primers varied from 74.5 to 88.7.

### 2.5. Real-Time Multiplex SG-PCR

Real-time multiplex SG-PCR and data analysis were performed for a total volume of 20 *μ*L using 96-well reaction plates and an ABI7500 or ABI7500 Fast Real-Time PCR system (Applied Biosystems), LightCycler 480 (Roche) or Thermal Cycler Dice Real-Time System (Takara, Japan). Each reaction tube contained 10 *μ*L of SYBR *Premix DimerEraser *(Takara, Japan), 0.4 *μ*L of ROX Reference Dye II (50×) (for ABI 7500 and ABI7500 Fast), 0.8 *μ*L (for ABI 7500 and ABI 7500 Fast), or 1.2 *μ*L (for LightCycler 480 and Thermal Cycler Dice) of PCR-grade H_2_O, each 1.2 *μ*L of a 10 *μ*M primer set for 3 target genes, 1.2 *μ*L of a 10 *μ*M IAC primer set, 2 *μ*L of IAC DNA, and 2 *μ*L of sample DNA in a 20 *μ*L PCR mixture. In each of 8 lines (12 wells per line) on a 96-well reaction plate, the samples were set as negative control (4 *μ*L of dH_2_O) in the 1st well, each 2 *μ*L of IAC, and dH_2_O in the 2nd well, each 2 *μ*L of IAC, and 1 out of 3 target positive controls in the 3rd to 5th wells, and 2 *μ*L of IAC and each 7 stool DNA samples in the 6th to 12th wells. The PCR amplicons resulting from foodborne pathogens and *Y. ruckeri* were used for the positive controls and IAC, respectively. The concentrations of positive control (equal 10^5^ to 10^6^ cfu/g) were adjusted to become the *C_t_* values to 17 to 21 by dilution of 10^3^- to 10^4^-fold with Easy Dilution (Takara, Japan) and two IACs (equal 10^1^ to 10^2^ cfu/g) were adjusted to become the *C_t_* values to 27 to 29 by dilution of 10^6^- to 10^7^-fold with Easy Dilution. The assay cycling profile was one cycle of 95°C for 30 s followed by 30 cycles of denaturation at 95°C for 5 s (3 s for ABI 7500 Fast), annealing at 55°C for 30 s (34 s for ABI 7500) and then 72°C for 30 s (34 s for ABI 7500), and a dissociation stage of 1 cycle at 95°C for 15 s, 60°C for 60 s, and 95°C for 15 s. The specificity of the reaction was found by the detection of the *Tm*s of the amplification products immediately after the last reaction cycle. These reactions were finished in 2 hours or less. Results were analyzed with SDS software provided with each real-time PCR system.

### 2.6. Multiplex Real-Time SG-PCR Analysis in 35 Foodborne Outbreaks

Multiplex real-time SG-PCR analysis of foodborne outbreak was experimentally tested using the ABI 7500 in Shimane (22 cases between 2002 and 2009), Fukuoka (3 cases between 2006 and 2009), and Shizuoka Prefecture 3cases on 2009), using ABI 7500 Fast in Fukuoka Prefecture (2 cases on 2009), using Thermal Cycler Dice Real Time System in Hokkaido (3 cases between 2008 and 2009), and using LightCycler 480 in Kumamoto Prefecture (2 cases on 2009) (Table 3). The DNA samples were extracted with the QIAamp DNA Stool Mini kit from patient fecal samples (within 1 hour) and were set on a 96-well reaction plate as described above. The samples before 2008 were used after 1 to 3 years store at −20°C. The multiplex PCR assay was evaluated with regard to detection (in 2 hours or less) of 24 specific genes of foodborne pathogens in 7 stool specimens. Each PCR product was generated with a different *Tm *curve among 4 *Tm *curves of PCR target gene products. These could all be resolved using each software and *Tm *curve analysis whenever target bacteria were present in the reaction well.

## 3. Results and Discussion

### 3.1. Noncompetitive IAC and Two IAC Primers

 In this study, the *Y. ruckeri* bacterium was successfully used as a noncompetitive IAC and for 2 pairs of IAC primer for routine detection of 24 target genes of foodborne pathogens. Using an IAC with real-time PCR detection is important to identify false negative results and to control for the presence of amplification inhibitors [26]. It is important to take into account that components of the sample or the competing microflora may influence the effectiveness of the PCR, especially by reducing the detection limit and producing false negative results. The consequences of false negative results in the detection of a pathogenic microorganism may potentially be life threatening [27]. The European Standardization Committee, in collaboration with International Standard Organization (ISO), has proposed a general guideline for PCR testing of foodborne pathogens that requires presence of IAC in the reaction mixture [28]. 

While some design approaches such as cloning require substantial technical skills, others can be done using basic PCR methodology. There are two main strategies for use of an IAC in a diagnostic real-time PCR assay. Their difference lies in whether the IAC is to be used competitively or noncompetitively [5]. By using the composite primer technique, the target and the competitive IAC are amplified with one common set of primers and under the same conditions and in the same real-time PCR tube. The competitive IAC method was used for TaqMan PCR to detect *S. enterica *[26, 29, 30], *E. coli* O157 [31], and *C. botulinum* [32] and real-time SG-PCR to detect *C. botulinum* [33]. However, these competitive IAC methods can lower the amplification efficiency, which results in a lower detection limit [5]. In the noncompetitive IAC method, the target and IAC are amplified using a different primer set for each. The disadvantage is that amplification of the IAC may not accurately reflect amplification of the target. This method was used for TaqMan PCR to detect *Campylobacter* spp. [9, 34], *B. cereus* [35], *C. botulinum *[36], and* V. parahaemolyticus* [7]. These assays were used for the detection of single target gene except for the four-target TaqMan multiplex PCR to detect *V. parahaemolyticus* [7]. Although the main advantage of the noncompetitive IAC method is that it can be used for many different assays in the same laboratory [5], we do not have a unique real-time PCR assay for the detection of almost all foodborne pathogens using universal IAC.

 Each previously described method for introduction of an IAC is limited due to primer competition or because it requires the presence of a specific substrate or organism. The new approach presented in this paper comprises a separate amplification of target DNA and noncompetitive IAC-DNA using each specific target primer set and two different IAC primer sets on the detection of each foodborne pathogens. The latter is based on 16S rRNA of *Y. ruckeri,* which is not found naturally in human stool and food samples. 

**Figure 1:**
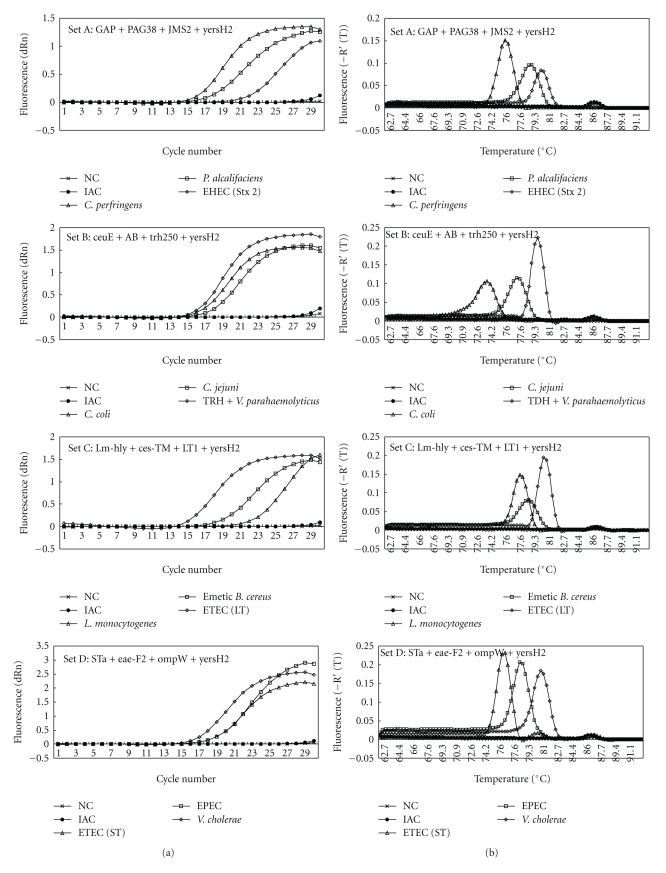

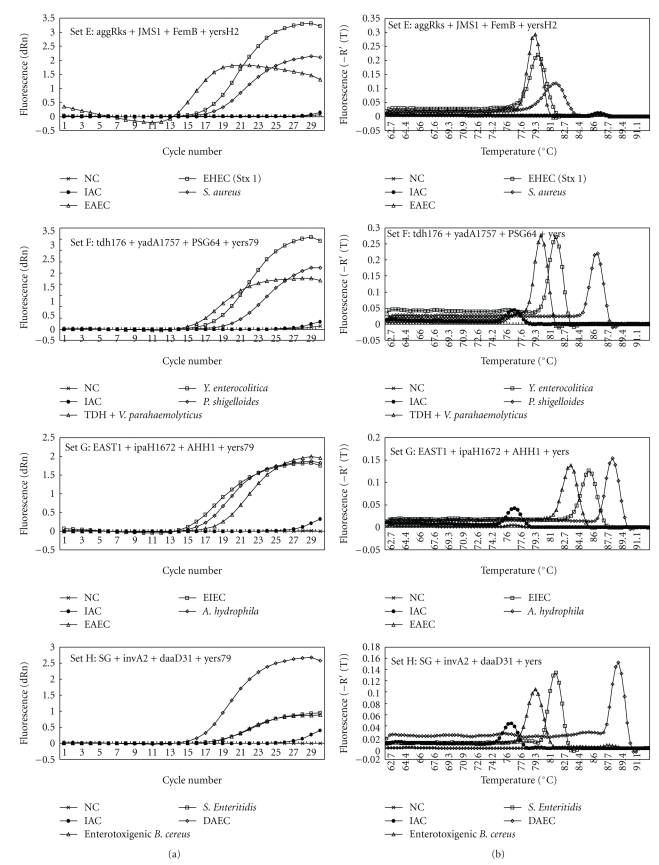
Amplification (a) and melting curve analysis (b) of 3 target genes of foodborne pathogens and IAC gene by primer sets A to H in multiplex real-time SG-PCR.

**Figure 2:**
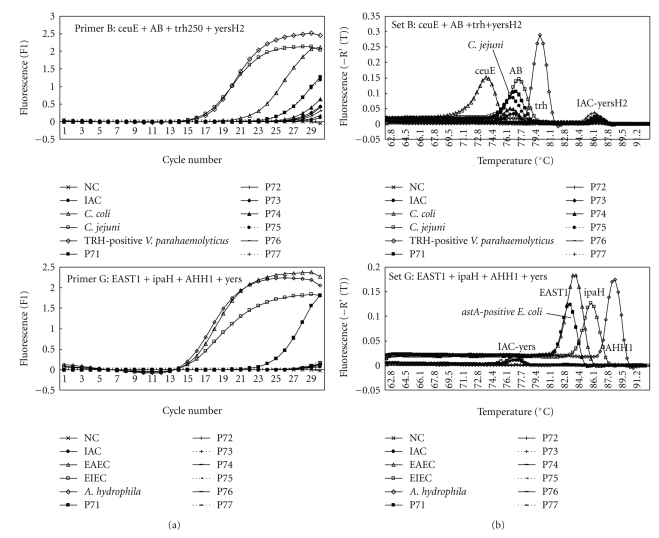
Melting curve analysis of multiplex real-time SG-PCR products from 7 stool samples in case 21 of a foodborne outbreak. Two panels show detection of target genes of foodborne pathogens by primer sets B and G, but it was not detected by the other 6 primer sets.

 The IAC primer *yersH2 *was used for detection of 15 target genes of foodborne pathogens which *Tm* values of amplicons were lower than 83°C and shown as primer sets A to E (described in the next section), and the IAC primer* yers* was used for detection of 9 target genes of foodborne pathogens which *Tm* values of amplicons were over than 80°C and shown as primer sets F to H (described in the next section). The IAC-specific low peak on a *Tm *curve analysis was present in all reaction tubes added with IAC and IAC primers and in all the negative results of target PCR in reaction tubes added with IAC primers (Figures 1 and 2).

### 3.2. Development of PCR Procedures for a Set of 8 Multiplex Assays

We developed the ultimately new PCR screening system for foodborne pathogens in stool specimens. One can simultaneously analyze 24 pathogenic or specific genes of foodborne pathogens in 7 stool specimens by using multiplex real-time SG-PCR containing IAC and 96-well reaction plate. Single or multiple real-time PCR assays were reported for detection of one species among foodborne pathogens, such as *E. coli* [11, 17, 18, 22], *Salmonella *[26, 28, 29], *C. jejuni *[13, 37], *V. cholerae *[19], *V. parahaemolyticus *[38], and *S. aureus *[21]. Comprehensive, rapid real-time SG-PCR procedures, which used 24 primer pairs for detection of 15 bacterial species including: 6 groups of *E. coli, *2 subgroups each of *B. cereus *and *V. parahaemolyticus,* and 2 primer pairs for an IAC, were developed using a set of 8 multiplex PCR assays with 3 primer pairs for foodborne pathogens and an IAC primer pair. Nineteen pairs of primers for foodborne pathogens were selected from earlier publications (Table 1), and 5 pairs of primers for *tdh *gene of TDH-positive *V. parahaemolyticus, yadA* gene of *Y. enterocolitica* and *Y. pseudotuberculosis, gyrB* gene of *P. shigelloides, ipaH* gene of EIEC and *Shigella *spp., and *daaD* gene of DAEC were constructed. This was done to make all 24 SG-PCR methods suitable for the same PCR conditions (an annealing temperature of 60°C). The sequence, target, PCR product size, threshold cycle (*C_t_*) values, and *Tm *values of 24 primer pairs for target genes and 2 primer pairs for IAC are listed in Table 1. The specificity of the PCR assay was confirmed on 659 strains listed in Table 2. The STa-F and STa-R primer pair could not detect *st* gene from 5 of 18* st*-positive ETEC strains. The ipaH1672-F and ipaH1761-R primer pair cross-reacts with *Shigella *spp. and EIEC. The SG-F and SG-R primer pair cross-reacts with enterotoxigenic and emetic *B. cereus. *As same as previous studies [4], the eae-F2 and eae-R primer pair cross-reacts with EPEC and EHEC, and the EAST-1S and EAST-1AS primer pair cross-reacts with EAEC and some strains of EPEC, ETEC, and DAEC. The yadA667-F and yadA851-R2 for *Yersinia *adhesion reacts with virulent *Y. enterocolitica *and *Y. pseudotuberculosis, *but not with nonpathogenic strains of *Yersinia *spp.

 A Foodborne Outbreak Investigation Report (http://www.mhlw.go.jp/topics/syokuchu/), by the Ministry of Health, Labor and Welfare, Japan, during 2005 to 2008, shows that 97% of foodborne outbreaks were caused by the following 7 species of foodborne pathogens:* C. jejuni* (56.5%), *S. enterica *(16.0%), TDH-positive *V. parahaemolyticus* (10.0%)*, S. aureus *(6.8%)*, C. perfringens *(3.4%), emetic* B. cereus* (2.0%), and EHEC (2.4%), and other virulent *E. coli* (2.1%) which include *astA*-positive *E. coli *which is a strain of *E. coli *that does not possess any diarrheagenic characteristics except the EAEC heat-stable toxin 1 (EAST1) gene and is frequently isolated in diarrhea outbreaks [39]. Each primer set was combined with 4 primer pairs designed for 1 of 8 main foodborne pathogens and were also designed for IAC and 2 of 16 target genes of other foodborne pathogens (Table 2). Particularly each primer for 8 main foodborne pathogens was carefully set in 8 different primer sets for keeping away from the presence of multiple primers for main foodborne pathogens in the same reaction well. Really the plural target genes were detected from 26 stool samples in 15 cases of foodborne outbreaks but these target genes were, respectively, detected from different reaction wells (Table 3). The fluorescent amplification curves and *Tm *curves of the multiplex SG-PCR products of the DNA of foodborne pathogens and IAC were shown in Figure 1. The *C_t_* values of the amplicons resulting from foodborne pathogens were 17 to 21 and those of IAC were 27 to 29. In each analysis, the *T_m_* distance was from 0.8 to 6.2°C among the target gene's products. Looking at the short variations among some *T_m_* distance in set C and set E, the presence of *ces *gene in set C could be decided altogether with the presence of *nheB *gene of* B. cereus *in set H and the presence of *stx1* gene in set E could be decided altogether with the presence of *eaeA* gene of EHEC in set D. The IAC-specific low peak was present in all samples with added IAC (C*_t_*: 27 to 29) using real-time SG-PCR analysis of the 24 target genes of primer sets A to E including primer *yersH2* and of primer sets F, G, and H including primer *yers*. IAC was certainly amplified in the IAC-only samples. These could be resolved in the ABI 7500 by using *Tm *curve analysis when a target bacterium was present in the reaction tube. The *Tm *values of PCR products of stool samples, including each foodborne pathogens, could be identified with that of control bacteria in the same run based on a *Tm *curve analysis.

### 3.3. Multiplex SG-PCR for Identification of the Causative Pathogens in Foodborne Outbreaks

In foodborne outbreaks, stool specimens from patients infected with enteric bacteria with acute severe disease may contain large numbers of causative bacterial species [2, 11]. In most cases of foodborne outbreak, we found that causative bacteria can be rapidly detected and that a presumptive diagnosis of the causative agent of foodborne outbreak could be made within 3 hours. We used a combination of the multiplex real-time SG-PCR assay with DNA extraction with the QIAamp DNA Stool Mini kit used for detection. Almost all bacterial pathogens are detectable in stool specimens at a concentration of 10^3^ to 10^4^ bacteria per g. This is because the concentration of DNA extracted from stool specimens using this DNA extraction kit was finally diluted to 6 × 10^4^-fold in the reaction mixture. The PCR sensitivity for bacteria inoculated in stool samples may be as low as the presence of 10 cells in the reaction well, as described in our previous report [2]. The real-time SG-PCR assay is a rapid, specific, and sensitive detection technique. The DNA extraction of 7 stool specimens with this DNA extraction kit was carried out within 1 hour. Then, the multiplex real-time SG-PCR assay was also carried out within 2 hours, and we could then specifically identify the products based on a *Tm *curve analysis. For example, Figure 2 shows those of case 21, in which *C. jejuni* and *astA*-positive *E. coli* strains were isolated from 4 and one of 7 stool specimens of symptomatic patients, respectively. Two panels show detection of target genes of foodborne pathogens by primer sets B and G, but it was not detected by the other 6 primer sets. In multiplex PCR analysis, the *C. jejuni*-specific gene and the *astA* gene were simultaneously detected by primer sets B and G from the same culture—positive stool specimens.

### 3.4. Identification of the Causative Pathogens in 35 Foodborne Outbreaks using Multiplex SG-PCR

Table 3 shows epidemiological and clinical investigations in 35 foodborne outbreaks (occurred between 2002 and 2009) examined by multiplex SG-PCR analysis in 5 different laboratories in 2009. DNA samples extracted from 2 to 7 feces of symptomatic patients were stored at −20°C until using. In 33 (94.3%) of 35 foodborne outbreak cases, the causative bacteria and/or some sporadic bacteria were comprehensive and simultaneously detected using multiplex SG-PCR from stool specimens. Moreover, the same reactions, which IAC-specific low peak was present in reaction tubes added with IAC and IAC primer *yersH2 *or *yers*, were observed. This demonstrated the absence of PCR inhibitor in DNA specimens extracted from patient stool specimens using this DNA extraction kit. In this study, it was confirmed that using IAC and 2 IAC primers with different *Tm* values was advantageous to allow elimination of false negative results in real-time SG-PCR for the detection of 24 target genes of foodborne pathogens. The results of multiplex real-time SG-PCR assay of 7 foodborne outbreaks were confirmed by the use of IAC and 2 IAC primers. The certain amplification of target genes and IAC in each multiplex PCR analysis demonstrated the usefulness of this multiplex real-time SG-PCR as reliable diagnostic PCR.

The target genes of 12 species of foodborne bacteria (*C. jejuni, E. coli, C. perfringens, S. aureus, Salmonella *spp*., V. parahaemolyticus, V. cholerae* non-O1*, B. cereus, P. alcalifaciens, P. shigelloides,* and* A. hydrophila*), which included 5 groups of *E. coli* (EHEC, EPEC, EAEC, ETEC, and *astA*-positive *E. coli*), were detected from 129 (64.8%) of 199 feces in 33 (94.3%) of 35 cases by multiplex SG-PCR, from 1 to 7 samples. Multiplex SG-PCR rapidly and accurately demonstrated that 11 (31.4%) of 35 cases were caused with a single foodborne pathogen such as *C. jejuni *(7 cases), *C. perfringens *(2 cases), *B. cereus *(1 case), and *S*. Enteritidis (1 case). There were also 19 (54.2%) cases with plural foodborne bacterial pathogens and 3 (2.9%) cases with foodborne bacterial pathogens (*astA*-positive *E. coli,* EHEC O:26, or *C. perfringens*) and norovirus. The causative pathogens had been isolated from 125 (62.8%) of 199 PCR examined samples and from 216 (56.7%) of 381 total samples in all 35 cases. Although the target genes of EPEC, EAEC, ETEC, *astA*-positive *E. coli, P. alcalifaciens,* and* A. hydrophila* were detected by SG-PCR, the isolation of these pathogens from the stool samples containing much normal *E. coli* flora was difficult. This analysis may be a very useful tool for the detection of these unusual pathogens which are generally difficult to isolate. We previously reported that the presence of any foodborne pathogens at more than 10^3^ CFU/g feces was certainly confirmed by melting curve analysis in duplex SG-PCR [2, 4]. In this multiplex PCR analysis including IAC, the presence of any foodborne pathogens at more than 10^5^ CFU/g feces was certainly confirmed in 40 (97.6%) of 41 samples by melting curve analysis, 10^4^ CFU/g feces was confirmed in 7 (63.6%) of 11 samples and 10^3^ CFU/g feces in 3 (50%) of 6 samples (Table 4). The sensitivity of this multiplex SG-PCR including IAC might became slightly lower than that of duplex SG-PCR (absent IAC), caused by the interference among 4 primer pairs including IAC primer in the same reaction well. In 2 cases (5.7%), in which *S. enteric *serovar Enteritidis was isolated by direct culture (unknown cfu) from one patient in case 23 and 10^4^ cfu/g of feces from 2 patients in case 26, the target gene of *Salmonella* was not detected by multiplex SG-PCR, because the sensitivity of invA2 primer may be slightly lower than those of other primers. The choice or design of more sensitive primer for the detection of *Salmonella* spp. is indispensable in future studies.

### 3.5. Usefulness of Multiplex SG-PCR for the Rapid Diagnostic Test in Foodborne Outbreaks

Systematically reviewing clinical implications, public health considerations, and cost-effectiveness of rapid diagnostic tests for detection and identification of bacterial intestinal pathogens in feces and food [1], economic modeling suggests that adoption of rapid test methods, especially for PCR, in combination with a routine culture is unlikely to be cost-effective, however, as the cost of rapid technologies decreases, total replacement with rapid technologies may be feasible. Despite the relatively poor quality of reporting of studies evaluating rapid detection methods, the reviewed evidence shows that PCR for *Campylobacter*, *Salmonella, *and *E. coli *O157 is potentially very successful in identifying pathogens. It is possibly detecting more than the numbers currently being reported using cultures. Less is known about the benefits of testing for *B. cereus*, *C. perfringens, *and *S. aureus*. This review pointed out that further investigation is needed on how clinical outcomes may be altered if test results are available more quickly and at greater precision than the current practice of using bacterial culture [1]. In the present study, simple and specific methods were established to detect comprehensive and simultaneously 24 specific genes of foodborne pathogens including main bacterial pathogens such as *Campylobacter*, *Salmonella, E. coli *O157, *B. cereus*, *C. perfringens, *and *S. aureus *in 7 stool specimens in a real-time SG-PCR assay using a 96-well reaction plate containing a universal noncompetitive IAC. The usefulness of this method for the rapid diagnostic tests was confirmed by the successful detection of causative bacteria in 33 foodborne outbreak cases.

In conclusion, the multiplex real-time method described here for simultaneous screening of 24 target genes of foodborne pathogens were comprehensive, rapid, inexpensive, highly selective, accurate, and demonstrated detection probability. Due to the use of IAC and 2 IAC primers, the assay is suitable for accurate and rapid diagnosis of almost all foodborne pathogens in stool specimens of foodborne outbreak outbreaks. In future studies, workers should improve the kit of multiplex real-time PCR and select more suitable primers for foodborne pathogens.

## Figures and Tables

**Table 1:** Eight sets of real-time multiplex PCR with 4 primer pairs for 3 target genes and an IAC gene prior to comprehensive and rapid analysis of foodborne outbreak.

Primer set		Species	Target gene	Primer name	Sequence (5′–3′)	Gene bank accession no.	Location	Product size (bp)	*T* *m* ^a^	*Tm* distance	Refer-ences
	∗^b^	*Clostridium perfringens*	*cpe*	GAP-11	GGTTCATTAATTGAAACTGGTG	X81849	583–604	154	75.8 ± 0.37		[10]
				GAP-12	AACGCCAATCATATAAATTACAGC		712–736			3.7	
A		*Providencia alcalifaciens*	*gyrB*	PAG38-F	TCTGCACGGTGTGGGTGTT	AJ300547	38–56	73	79.5 ± 0.79		[2]
				PAG110-R	ACCGTCACGGCGGATTACT		110–92			1	
		EHEC (Stx 2)	*Stx2*	JMS2-F	CGACCCCTCTTGAACATA	EF441616	140–157	108	80.5 ± 0.76		[11]
				JMS2-R	GATAGACATCAAGCCCTCGT		228–247				

		*Campylobacter coli*	*ceuE*	ceuE-For	CAAGTACTGCAATAAAAACTAGCACTACG	X88849	2777–2805	72	73.7 ± 0.43		[12]
				ceuE-Rev	AGCTATCACCCTCATCACTCATACTAATAG		2848–2819			4	
B	∗	*Campylobacter jejuni*	specific	AB-F	CTGAATTTGATACCTTAAGTGCAGC	AL111168	381121–	86	77.7 ± 0.96		[13]
				AB-R	AGGCACGCCTAAACCTATAGCT		381185–			1.9	
		TRH-positive *Vibrio parahaemolyticus *	*trh*	trh250-F	GGCTCAAAATGGTTAAGCG	AY742213	705–687	250	79.6 ± 0.21		[14]
				trh250-R	CATTTCCGCTCTCATATGC		456–474				

		*Listeria monocytogenes*	*hly*	Lm-hly-F	GGGAAATCTGTCTCAGGTGATGT	AF253320	973–995	106	77.4 ± 0.78		[15]
				Lm-hly-R	CGATGATTTGAACTTCATCTTTTGC		1078–1054			1.5	
C	∗	Emetic *Bacillus cereus *	*ces*	ces-TM-F	GATGTTTGCGACGATGCAA	DQ360825	8689–8707	65	78.9 ± 0.87		[4]
				ces-TM-R	CTTTCGGCGTGATACCCATT		8734–8793			1.6	
		ETEC (LT)	*lt*	LT-1	TTACGGCGTTACTATCCTCTCTA	X83966	233–255	275	80.5 ± 0.21		[16]
				LT-2	GGTCTCGGTCAGATATGTGATTC		507–485				

		ETEC (ST)	*st*	STa-F	GCTAATGTTGGCAATTTTTATTTCTGTA	M25607	294–321	190	77.1 ± 0.55		[17]
				STa-R	AGGATTACAACAAAGTTCACAGCAGTAA		456–483			1.7	
D	∗	EHEC and EPEC	*eaeA*	eae-F2	CATTGATCAGGATTTTTCTGGTGATA	Z11541	899–924	106	78.8 ± 0.54		[18]
				eae-R	CTCATGCGGAAATAGCCGTTA		979–1000			2.6	
		*V. cholerae*	ompW	ompW-F	AACATCCGTGGATTTGGCATCTG	X51948	675–692	89	81.4 ± 0.69		[19]
				ompW-R	GCTGGTTCCTCAACGCTTCTG		741–763				

		EAEC	*aggR*	aggRks1	GTATACACAAAAGAAGGAAGC	Z18751	18–38	254	79.2 ± 0.25		[20]
				aggRKas2	ACAGAATCGTCAGCATCAGC		170–151			0.8	
E		EHEC (Stx 1)	*Stx1*	JMS1-F	GTCACAGTAACAAACCGTAACA	EF441598	509–488	95	80.0 ± 0.72		[11]
				JMS1-R	TCGTTGACTACTTCTTATCTGGA		415–437			1.6	
	∗	S*taphylococcus aureus *	*femB*	FemB-fw	AATTAACGAAATGGGCAGAAACA	AF106850	277–299	93	81.6 ± 0.62		[21]
				FemB-rv	TGCGCAACACCCTGAACTT		351–370				
	∗	TDH-positive *Vibrio parahaemolyticus *	tdh	tdh-F176	TCCATCTGTCCCTTTTCCTG	X54341	176–195	247	80.1 ± 0.22		This study
				tdh-R422	AGACACCGCTGCCATTGTAT		422–403			1.9	
F		*Y. enterocolitica *and *Y. pseudotuberculosis *	*yadA*	yadA-F1757	ACGAGTTGACAAAGGTTTAGCC	X13882	1757–1778	129	82.0 ± 0.38		This study
				yadA-R1885	GAACCAACCGCTAATGCCTGA		1885–1865			4.3	
		*Plesiomonas shigelloides*	*gyrB*	PSG-F64	TTAACGCCCTGTCGGATAAG	AJ300545	64–83	250	86.3 ± 0.26		This study
				PSG-R313	TCGAGCAGATGAATCGACAC		313–294				

	∗	EAEC	*astA*	EAST-1-S	GCCATCAACACAGTATATCC	L11241	63–82	106	83.7 ± 0.88		[22]
				EAST-AS	GAGTGACGGCTTTGTAGTCC		148–168			1.5	
G		EIEC and *Shigella *spp.	*ipaH*	ipaH1672-F	CTCTCAGAGGGTGGCTGACC	M32063	1672–1691	90	85.2 ± 0.31		This study
				ipaH1761-R	TCACGCATCACCTGTGCA		1761–1743			3.1	
		*Aeromonas hydrophila*	*ahh1*	AHH1-F	GCCGAGCGCCCAGAAGGTGAGTT	CP000462	1653360–82	130	88.3 ± 0.48		[23]
				AHH1-R	GAGCGGCTGGATGCGGTTGT		1653473–92				

		Enterotoxigenic *B. cereus *	*nheB*	SG-F3	GCACTTATGGCAGTATTTGCAGC	DQ153257	2101–2123	152	80.5 ± 0.84		[24]
				SG-R3	GCATCTTTTAAGCCTTCTGGTC		2231–2252			1.9	
H	*∗*	*Salmonella *spp.	*invA*	invA2-F	GAT TCT GGT ACT AAT GGT GAT GAT C	M90846	132–156	288	82.4 ± 0.28		[25]
				invA2-R	GCCAGGCT AT CGCCAAT AAC		419–400			6.2	
		DAEC	*daaD*	daaD-F31	GTCACCTGCGGGATGTTACT	AF233530	31–50	233	88.6 ± 0.32		This study
				daaD-R263	AGCTCATGACGACCATCCTT		263–244				

IAC for sets A-E IAC for sets F,G,H		*Yersinia ruckeri*	*16S rRNA*	yersH2-F	GGCTCACCTAGGCGACGA	X75275	245–262	211	86.1 ± 0.53		This study
				yersH2-R	TCAGTGCTATTAACACTTAACCCTTCC		455–429				
		*Yersinia ruckeri*	*16S rRNA*	yers-F	GGAGGAAGGGTTAAGTGTTA	X75275	426–443	68	77.2 ± 0.53		[9]
				yers-R	GAGTTAGCCGGTGCTTCTT		475–493				

^a^Values represent means ± standard deviations of 15 to 60 tests. ^b^Eight main foodborne bacteria.

**Table 2:** 659 bacterial strains assayed by real-time PCR.

Bacterial	Presence of		Number of strain	PCR positive result with each primer set^h^																									
strain	PCR target	Control		eae	JM	JM	LT	STa	agg	EAS	daa	ipa	inv	ya	PS	PA	A	ceu	AH	omp	tdh	trh	GAP	Lm-	Fe	ces	SG	yers	yersH2
	gene^a^	strain			S1	S2			R	T	D	H	A	dA	G	G	B	E	H1	W				hly	mB				
*Escherichia coli*—STEC	*eae, stx1, stx2 *	SE02007	20	20	20	20	−	−	−	−	−	−	−	−	−	−	−	−	−	−	−	−	−	−	−	−	−	−	−
	*eae, stx1*		15	15	15	−	−	−	−	−	−	−	−	−	−	−	−	−	−	−	−	−	−	−	−	−	−	−	−
	*eae, stx2*		7	7	−	7	−	−	−	−	−	−	−	−	−	−	−	−	−	−	−	−	−	−	−	−	−	−	−
	*stx1, stx2*		2	−	2	2	−	−	−	−	−	−	−	−	−	−	−	−	−	−	−	−	−	−	−	−	−	−	−
	*stx2*		1	−	−	1	−	−	−	−	−	−	−	−	−	−	−	−	−	−	−	−	−	−	−	−	−	−	−
*E. coli*—EPEC	*eae*	EC2736^b^	3	3	−	−	−	−	−	−	−	−	−	−	−	−	−	−	−	−	−	−	−	−	−	−	−	−	−
	*eae, astA*		5	5	−	−	−	−	−	5	−	−	−	−	−	−	−	−	−	−	−	−	−	−	−	−	−	−	−
*E. coli*—ETEC	*LT*		3	−	−	−	3	−	−	−	−	−	−	−	−	−	−	−	−	−	−	−	−	−	−	−	−	−	−
	*ST*		9	−	−	−	−	8	−	−	−	−	−	−	−	−	−	−	−	−	−	−	−	−	−	−	−	−	−
	*astA, LT*		1	−	−	−	1	−	−	1	−	−	−	−	−	−	−	−	−	−	−	−	−	−	−	−	−	−	−
	*astA, LT,ST*	EC3515^b^	2	−	−	−	2	2	−	2	−	−	−	−	−	−	−	−	−	−	−	−	−	−	−	−	−	−	−
	*astA, ST*		7	−	−	−	−	3	−	7	−	−	−	−	−	−	−	−	−	−	−	−	−	−	−	−	−	−	−
*E. coli*—ETEC	*astA, aggR*	EC4131^b^	8	−	−	−	−	−	8	8	−	−	−	−	−	−	−	−	−	−	−	−	−	−	−	−	−	−	−
	*aggR*		26	−	−	−	−	−	26	−	−	−	−	−	−	−	−	−	−	−	−	−	−	−	−	−	−	−	−
	*astA*		30	−	−	−	−	−	−	30	−	−	−	−	−	−	−	−	−	−	−	−	−	−	−	−	−	−	−
*E. coli*—DAEC	*daaD, astA*		2	−	−	−	−	−	−	2	2	−	−	−	−	−	−	−	−	−	−	−	−	−	−	−	−	−	−
	*daaD*	KI2214^c^	2	−	−	−	−	−	−	−	2	−	−	−	−	−	−	−	−	−	−	−	−	−	−	−	−	−	−
*E. coli*—EIEC	*ipaH*	EA32^d^	5	−	−	−	−	−	−	−	−	5	−	−	−	−	−	−	−	−	−	−	−	−	−	−	−	−	−
*Shigella *spp.	*ipaH*	I00031	38	−	−	−	−	−	−	−	−	38	−	−	−	−	−	−	−	−	−	−	−	−	−	−	−	−	−
*Salmonella *spp.	*invA*	Sal2339	31	−	−	−	−	−	−	−	−	−	31	−	−	−	−	−	−	−	−	−	−	−	−	−	−	−	−
*Yersinia enterocolitica*	*yadA*	Pa241	28	−	−	−	−	−	−	−	−	−	−	28	−	−	−	−	−	−	−	−	−	−	−	−	−	−	−
*Y. pseudotuberculosis*	*yadA*	SP988	27	−	−	−	−	−	−	−	−	−	−	27	−	−	−	−	−	−	−	−	−	−	−	−	−	−	−
*Plesiomonas shigelloides*	*gyrB*	NIID123^e^	4	−	−	−	−	−	−	−	−	−	−	−	4	−	−	−	−	−	−	−	−	−	−	−	−	−	−
*Providencia alcalifaciens*	*gyrB*	NIID124^e^	8	−	−	−	−	−	−	−	−	−	−	−	−	8	−	−	−	−	−	−	−	−	−	−	−	−	−
*Campylobacter jejuni*	specific gene	SC01	43	−	−	−	−	−	−	−	−	−	−	−	−	−	43	−	−	−	−	−	−	−	−	−	−	−	−
*Campylobacter coli*	*ceuE*	SC009	13	−	−	−	−	−	−	−	−	−	−	−	−	−	−	13	−	−	−	−	−	−	−	−	−	−	−
*Aeromonas hydrophila*	*ahh1*	AT CC7966	45	−	−	−	−	−	−	−	−	−	−	−	−	−	−	−	45	−	−	−	−	−	−	−	−	−	−
*Vibrio cholerae*	*OmpW*	AT CC14035	17	−	−	−	−	−	−	−	−	−	−	−	−	−	−	−	−	17	−	−	−	−	−	−	−	−	−
*Vibrio parahaemolyticus*	*tdh*	SVP02	48	−	−	−	−	−	−	−	−	−	−	−	−	−	−	−	−	−	48	−	−	−	−	−	−	−	−
	*tdh, trh*		2	−	−	−	−	−	−	−	−	−	−	−	−	−	−	−	−	−	2	2	−	−	−	−	−	−	−
	*trh*	NIIDk4^e^	35	−	−	−	−	−	−	−	−	−	−	−	−	−	−	−	−	−	−	35	−	−	−	−	−	−	−
*Clostridium perfringens*	*cpe*	H2^f^	41	−	−	−	−	−	−	−	−	−	−	−	−	−	−	−	−	−	−	−	41	−	−	−	−	−	−
*Listeria monocytogenes*	*hly*	Scott A	46	−	−	−	−	−	−	−	−	−	−	−	−	−	−	−	−	−	−	−	−	46	−	−	−	−	−
*Staphylococcus aureus*	*FemB*	SS05	35	−	−	−	−	−	−	−	−	−	−	−	−	−	−	−	−	−	−	−	−	−	35	−	−	−	−
Emetic *Bacillus cereus *	*ces, nheB*	No.127^g^	24	−	−	−	−	−	−	−	−	−	−	−	−	−	−	−	−	−	−	−	−	−	−	24	24	−	−
Enterotoxgenic *B. cereus *	*nheB*	No.1^g^	25	−	−	−	−	−	−	−	−	−	−	−	−	−	−	−	−	−	−	−	−	−	−	−	25	−	−
*Yersinia ruckeri*	16S rRNA	JCM15110	1	−	−	−	−	−	−	−	−	−	−	−	−	−	−	−	−	−	−	−	−	−	−	−	−	1	1

^a^Presence of PCR target gene was determined by another conventional PCR primer sets before this test.

^b^Strain kindly donated by J. Yatsuyanagi, Akita Prefectural Institute of Public Health (Akita, Japan) ^c^ *K*. Ito, National Institute of Infectious Disease (Tokyo, Japan)., ^d^ *K*. Sugiyama, Shizuoka Prefectural Institute of Public Health (Shizuoka, Japan), ^e^ *E*. Arakawa, National Institute of Infectious Disease (Tokyo, Japan), ^f^ *S*. Kaneko, Tokyo Metropolitan Institute of Public Health (Tokyo, Japan), ^g^ *S*. Ueda, Kagawa Nutrition University (Saitama, Japan) ^h^number positive result; − negative result. See Table 2 for primer sets.

**Table 3:** Epidemiological investigations in 21 foodborne outbreaks examined by SG-PCR and bacteriological cultures in Shimane Prefecture, Japan.

							Real-time PCR		Isolation		
Case No.^a^	Date occurred (day/mo/yr)	Days for examination after occurrence	Infected group	Source of infection (suspected source)	No. of patients/total	No. of examined patients	Target genes	No. of positive/examined samples	Causative pathogens	No. of positive/PCRexamined samples	No. of positive/total examined samples
1	4-Oct-02	6	School excursion in a mountain area	Stream water	23/33	22	*eaeA *and *astA *	1/7	EPEC	1/7	5/22
							*eaeA*	3/7	*astA*-positive *E. coli *	1/7	4/22
							*astA*	2/7			
2	03-Sep-03	3	Protective care school	Catering box lunch	22/46	10	*astA*	2/7	*astA*-positive *E. coli *	2/7	3/10
							*eaeA*	1/7			
							*gyrB *of *P. a *and *ahh1 *	1/7			
3	01-Oct-03	2	Celebration in a company	Catering box lunch	437/1354	12	*cpe*	5/7	*C. perfringens *O:13, O:16	6/7	10/12
4	11-Jun-04	6	Camping group of high school	Grilled meat (beef, bovine intestinal)	4/8	4	Specific gene of *C. j *	5/7	*C. jejuni*	5/7	5/8
5	12,13-Jun-04	6 ~ 7	9 citizen groups in Chophouse	Grilled meat (beef, bovine intestinal)	30/UN	12	Specific gene of *C. j *	4/7	*C. jejuni*	5/7	10/12
6	17-Jun-04	5	Cooking practise in a high school	Shelf-cooked lunch (salada mixed)	31/41	20	Specific gene of *C. j *	4/7	*C. jejuni*	6/7	17/20
7	07-Jul-04	1	Citizen in Chinese restaurant	Fried rice	6/6	6	*ces *and *nheB *	2/6	*B. cereus*	2/6	2/6
8	11-Oct-04	3	Sport club in a high school	Shelf-cooked lunch	26/47	6	*cpe*	2/6	*C. perfringens *O:16, OUT	3/6	4/6
							*astA *and *st *	1/6			
9	5~7-Nov-04	5 ~ 7	Restaurant	Unknown	5	5	Specific gene of *C. j *	2/5	*C. jejuni*	2/5	2/5
10	Unknown	Several days (19-Jan-05)	Nursery	Unknown	24/73	22	*eaeA *and *stx1 *	4/7	EHEC O26 [Norovirus	5/7	8/2220/22]
11	28~30-Sep-05	1 ~ 3	Prisoners in a prison	Shelf-cooked meal	113/600	61	*astA *and* cpe *	1/7	*astA*-positive *E. coli *	7/7	41/46
							*astA*	6/7	(*C. perfringens: *sporadic case	1/7	1/46)
12	2~6-Oct-05	1 ~ 5	Elementary and high school children	Unknown (School lunch)	39/94	39	*astA*, *aggR* and Specific gene of *C. j *	1/6	(*C. jejuni: *sporadic case	1/6	1/16)
							*astA *and* aggR *	1/6			
							*astA*	4/6			
13	28~30-May-06	0 ~ 2	Citizens at restaurant	Lunch (pilaf and scrambled agg)	27/34	27	*femB*	1/5	*S. aureus*	2/5	4/8
							*astA*	1/5			
14	4-Jul-06	0	Boarder of high school	Catering box lunch	34/51	34	*cpe*	7/7	*C. perfringens*	7/7	19/50
15	16-Aug-06	1	Citizens at restaurant	Fried rice	15/34	15	*ces *and *nheB *	1/4	*B. cereus*	2/4	2/4
							*aggR*	1/4			
16	23~29-Aug-06	2 ~ 8	Boarder of training high school	Supper (contaminated sliced cabbage)	19/43	18	*astA *and Specific gene of *C. j *	5/7	*C. jejuni*	6/7	9/14
							Specific gene of *C. j *	1/7			
17	2-Sep-06	3	Citizens in Buddhist service	Catering box lunch	14/49	6	*tdh*	3/6	*V. parahaemolyticus*	3/6	3/6
							Specific gene of *C. j *	2/6			
							*St*	1/6			
18	22-Dec-06	5	Citizens at restaurant	Supper (chicken)	12/12	9	Specific gene of *C. j *	4/7	*C. jejuni*	4/7	4/9
19	21-Oct-07	1	Citizens at restaurant	Supper	7/13	7	*gyrB *of *P. s *	2/5	*P. shigelloides*	2/5	2/5
							*eaeA*	1/5			
20	4-Jul-07	6	Citizens at restaurant	Supper (chicken)	7/11	3	Specific gene of *C. j *	1/2	*C. jejuni*	1/2	2/3
21	29-Nov-07	1	Citizens at restaurant	Supper (raw chicken liver)	8/13	6	*astA *and Specific gene of *C. j *	1/7	*C. jejuni*	4/7	4/7
							Specific gene of *C. j *	3/7	(*astA-positive E. coli *	1/7)	
22	28-Mar-08	5	Citizens at restaurant	Sushi	2/7	4	Specific gene of *C. j *	2/4	*C. jejuni*	2/4	2/7
23	16-Oct-08	2	workmate	home-made vinegared rice with thin strips of egg	13/15	4	Not detected	0/2	*Salmonella *Enteritidis	1/2	3/4
24	11-Jul-09	6	Citizens at restaurant	Grilled meat (beef, bovine intestinalmeat, raw liver)	4/4	4	Specific gene of *C. j *	3/4	*C. jejuni*	3/4	3/4
25	28-Jul-09	2	Citizens at restaurant	Unknown	13/44	5	*eaeA *and *astA *	1/5	STEC O63 (*stx2f*)	1/5	1/5
							*astA*	1/5			
26	25-Aug-09	13	customers of supermarket	Bowl of rice topped with deep-fried poak	4/4	2	Not detected	0/2	*Salmonella *Enteritidis	2/2	2/2
27	29-Sep-09	3 ~ 7	Employee of restaurant after EHEC O157 outbreak	Unknown	Not tested	7	*eaeA, stx1 *and *stx2 *	2/7	EHEC O157	4/7	4/7
							*eaeA *and *astA *	1/7			
							*astA*	3/7			
28	1-Jun-08	3	Staff of public services	Catering box lunch	171/296	17	*cpe*	7/7	*C. perfringens*	7/7	16/17
29	20-Aug-09	1	Citizens stayed in a hotel	Box lunch served by the hotel	11/21	9	*femB*	4/4	*S. aureus*	4/4	6/9
30	21-Sep-08	4	Citizens	Unknown	9/16	4	Specific gene of *C.j *	4/4	*C. jejuni*	4/4	4/4
31	14-Jun-09	1 ~ 2	Hospital	Supper in hospital	34/148	7	*cpe *and *femB *	1/7	*C. perfringens*	5/7	5/7
							*cpe*	4/7	[Norovirus	2/7]	
32	21-Aug-09	5	Citizens at restaurant	Supper	7/10	3	*astA *and Specific gene of *C. j *	1/3	*C. jejuni*	1/3	1/3
							*astA*	1/3			
33	14-Nov-09	5 ~ 6	Citizens at restaurant	Supper	9/15	7	*astA *and Specific gene of *C. j *	2/7	*C. jejuni*	6/7	6/7
							*femB *and Specific gene of *C. j *	1/7			
							Specific gene of C. *j *	3/7			
34	15-Aug-09	2 ~ 3	School excursion	Supper (potato salada) in a hotel	32/73		*tdh*	1/7	*V. parahaemolyticus*	1/7	2/7
							*ompW*	1/7	*V. p *and *V. cholerae *non-O1	1/7	1/7
35	16-Sep-09	9	Citizens in Buddhist service	Catering box lunch	25/43		*invA*	3/7	*S. *Enteritidis	4/7	4/7

Total							Gene of main pathogen	129/199	Main pathogen	125/199	216/381
								64.8%		62.8%	56.7%

^a^Analysis was tested using the ABI 7500 in Shimane (cases 1 to 22), Fukuoka (cases 23 to 25), and Shizuoka Prefecture (cases 31 to 33); ABI 7500 Fast in Fukuoka Prefecture (cases 26 and 27), Thermal Cycler Dice Real-Time System in Hokkaido (cases 28 to 30), and Light Cycler 480 in Kumamoto Prefecture (cases 34 and 35).

**Table 4:** The relationship between PCR detection and CFU in 15 foodborne outbreak cases by viable cell counting.

		Multiplex SG-PCR negative samples						Multiplex SG-PCR positive samples								
Case	Causative foodborne pathogens	Number of samples						Number of samples								
		Total	log 10 cfu/g by viable cell counting					Total	Log 10 cfu/g by viable cell counting							
			0	2	3	4	5		0	2	3	4	5	6	7	8
3	*C. perfringens*	2	2					5					1	3		1
6	*C. jejuni*	3	2		1			4				1	3			
8	*C. perfringens*	4	2		1	1		2				1	1			
9	*C. jejuni*	3	3					2				2				
10	EHEC O26	3	2		1			4			1			1	1	1
11	*astA*-positive *E. coli *	0						7						1		6
13	*S. aureus*	4	3			1		1				1				
14	*C. perfringens*	0						7					1	3	3	
17	*B. cereus*	3	3					1				1				
18	*C. jejuni*	1					1	5					1		2	2
20	*C. jejuni*	3	3					4			1		3			
19	*P. shigelloides *	3	3					2					2			
	*astA*-positive *E. coli *	4	4					1				1				
21	*C. jejuni*	3	3					4					3	1		
26	*S. enterica *serovar Enteritidis	2				2		0								
27	EHEC O157	5	5					2		1	1					

	Total	43	35	0	3	4	1	51	0	1	3	7	15	9	6	10
